# Reliable novelty: New should not trump true

**DOI:** 10.1371/journal.pbio.3000117

**Published:** 2019-02-12

**Authors:** Björn Brembs

**Affiliations:** Universität Regensburg, Institut für Zoologie, Neurogenetik, Regensburg, Germany

## Abstract

Although a case can be made for rewarding scientists for risky, novel science rather than for incremental, reliable science, novelty without reliability ceases to be science. The currently available evidence suggests that the most prestigious journals are no better at detecting unreliable science than other journals. In fact, some of the most convincing studies show a negative correlation, with the most prestigious journals publishing the least reliable science. With the credibility of science increasingly under siege, how much longer can we afford to reward novelty at the expense of reliability? Here, I argue for replacing the legacy journals with a modern information infrastructure that is governed by scholars. This infrastructure would allow renewed focus on scientific reliability, with improved sort, filter, and discovery functionalities, at massive cost savings. If these savings were invested in additional infrastructure for research data and scientific code and/or software, scientific reliability would receive additional support, and funding woes—for, e.g., biological databases—would be a concern of the past.

## Abstract 2

This Perspective article asserts that the most prestigious journals publish the least reliable science, and asks how long we can afford to reward scientists for publishing there.

## Journal rank and confirmation bias

All scientists implicitly, and most explicitly, expect scientific breakthroughs to be published in the most prestigious journals. After all, do we not all send our most significant discoveries to exactly these journals? Consequently, we wonder at every instance of reading or discussing a less-than-stellar publication in a top-notch journal how it could get past the gatekeepers when they had such a wealth of outstanding research (perhaps one’s own among them) to choose from? Conversely, we shrug our shoulders when a similarly substandard article appears in a lesser journal: “Of course this rag would publish something like this.”

These hypothetical but no doubt very common sentiments are a prime example of confirmation bias: we tend to question and scrutinize unexpected events more than expected ones. In this case, we expect high-ranking journals to publish work that conforms to our own subjective standards of quality, and we wonder how anybody could not share our standards and publish certain articles we consider beneath our own standards. Because we develop professionally within a hierarchical ranking of journals, our expectations for what the content at each tier ought to look like vary to such an extent that some even perform their peer-reviews differently for journals from different tiers of this hierarchy. In this way, we constantly affirm our own expectations based on the strangely circular logic that “bad” publications are rare in top journals and common in lesser journals and that this is precisely what constitutes this hierarchy.

But is our subjective hierarchy accurately reflecting the content of each journal? Can we trust our own judgement about our journals? After all, many an experiment have yielded counterintuitive results [1–3], and the scientific method has shown us time and time again how misleading confirmation bias can be [4–7]. How can we test whether journal rank as we use it today is based on evidence? As in any scientific endeavor, there are several potential paths to answering this question. At least two main options readily spring to mind here: First, one can test whether the quantitative way journal rank itself is established today can be related to any notions of “quality.” The other is to simply take current journal rankings as a measure for prestige and search for measures of “quality” that correlate with the prestige.

## Establishing journal rank

Although there are numerous mathematical methods with which one can rank journals, the one single metric that dominates the market is Clarivate Analytics’ impact factor (IF). Over the decades, the numerous flaws in deriving this particular rank have been extensively covered. In brief, it suffers from three main flaws, together with a myriad of lesser flaws too numerous to recount here [8]. The three main flaws are that (a) the IF, purported to be calculated is, in fact, negotiated and this process has been publicly documented for decades [8–18] and was never disputed; (b) even if the IF were calculated, the published figures are not reproducible, even with the data from the corporation [19]; and (c) even if the IF were calculated and the results reproducible, the way in which it is calculated involves computing the arithmetic mean from highly left-skewed distributions [10,18,20–25], an obvious mathematical mistake.

Taken together, this body of evidence alone ought to rule out using IF for anything important. Indeed, the case has been made that scholars using it ought to be shamed for their incompetence [26]. However, one need not even look at how the IF is derived and published to come to the conclusion that there is little in the metric itself that bears any relation to any notion of whatever one may define as “quality.” Nominally, the IF is based on citations, but do citations even reflect a notion of quality? At first glance, one may be tempted to argue that only “good” work gets cited; but when even retracted, mostly fraudulent publications continue to get cited [27–29], this argument breaks down. Moreover, the number of citations is correlated with the size of the field as more authors write more papers that can cite any given paper [30,31]. Finally, citation practice is highly field specific, such that some fields have developed a practice of citing few and others of citing many previous works. Much like any other human behavior, many of the main factors influencing citation behavior are historical, psychological, and highly interconnected [69]. In other words, even if the IF were capturing citations flawlessly (which it is far from achieving), it would still not be considered a measure of quality rather than, at best, a weak, noisy signal of attention and field size.

Therefore, it is fair to exclude using this process of ranking journals itself as justification to assign different tiers of “quality” to different tiers of journals.

## Testing journal rank

### In search of quantifiable aspects of quality

It is a noteworthy discovery in and of itself that a number so flawed as the IF nevertheless correlates with anything, let alone exceedingly well with scholars’ subjective notion of journal prestige [32–36]. Due to this correlation between IF and subjective prestige rank, the IF lends itself as a tool to test several quantifiable aspects of quality and to see how well the hierarchy of prestige stands up against the scientific method.

According to a quote attributed to Albert Einstein, “Not everything that can be counted counts and not everything that counts can be counted.” Whether a publication is considered “good” depends on a number of variables. Among the most frequently cited is novelty, i.e., that the publication in question constitutes a discovery not made before and a significant scientific advancement. However, novelty alone is a questionable aspect of quality long before one attempts to quantify it. Whether a publication is novel depends on the knowledge, and thus perspective, of the reader. Similarly, what constitutes a significant advancement is highly subjective as well. For these reasons, a focus on novelty incentivizes authors, likely against their better knowledge, to make their work appear more novel, e.g., by using the word “novel” more often [37] or by leaving out references to prior work—a common practice that some journals seem to openly endorse [38]. Finally, table-top cold fusion, arsenic in DNA, or the purported link between the MMR vaccine and autism were at least as novel as the discovery of CRISPR gene scissors, gravitational waves, or place cells, and yet most would agree that there is an important enough distinction between the former group of “discoveries” and the latter, which justifies not treating them equivalently. In other words, novelty alone is useless as a signal of quality. Of course, if a discovery is truly novel, it cannot yet have been reproduced. Therefore, any journal rank that aspires to capture quality beyond mere novelty must be able to distinguish between submitted, novel manuscripts of the former, unreliable type and the second, reliable kind before actual replications have been attempted. Is our system of ranked journals up to this task? Given that we all send our most novel work to the best journals, are these top journals indeed able to separate the novel, reliable wheat from the novel, unreliable chaff?

In many fields, it will be nigh impossible to objectively and/or at least semi-automatically quantify many important aspects of the work. However, in the experimental sciences, luckily, there are aspects of reliability and accuracy that can be quantified objectively and compared across large numbers of articles and journals.

### The evidence against our notion of prestige

For instance, crystallographers quantify the quality and accuracy of computer models derived from experimental work in structural biology and chemistry by comparing the computer models to established properties of the substance’s constituencies. They use bond distances, angles, and other factors to derive a difference score that measures how far away a given model is from being perfectly accurate. Averaging thousands of such models over the journals they have been published in, prestigious journals such as *Cell*, *Molecular Cell*, *Nature*, *EMBO Journal*, and *Science* publish significantly substandard models of such structures [39].

Such prestigious journals have also been found to publish exaggerated effect sizes with lower than necessary sample sizes in single gene association studies for psychiatric disorders [40]. Overall statistical power has been found to be weak across the biomedical and psychological sciences [41–44], indicating an overall low reliability for these fields. Statistical power was found to be at best uncorrelated with journal rank [8], or it correlated negatively, i.e., publications in higher-ranking journals report a lower statistical power [42,44].

Animal disease models are subjected to similar procedures as clinical trials in humans to evaluate the effectiveness of the treatments. Clearly, only the highest standards of scientific rigor should apply to such experiments. Among the most basic standards are the randomized assignment of individuals to treatment and control groups and the blind assessment of the outcome. Analyzing the reporting of randomization and blinding in the methods sections, it becomes clear that not only is it rare that these basic procedures are reported, authors at high-ranking journals are worse at it than lesser journals. Therefore, at best, authors of publications in high-ranking journals are sloppier in reporting their methods than their counterparts in less prestigious journals [45]. At worst, they adhere less to basic notions of good experimental design.

Sloppiness may also be attributed whenever discrepancies can be found between the actual results of a study and what is reported in the publication. For instance, gene symbols and accession numbers may inadvertently be converted into dates or floating point numbers when -omics researchers copy and paste their results into Microsoft Excel spreadsheets without tedious error correction by hand. This is a rather common error, but it is noteworthy that the incidence of such errors is higher in more prestigious journals [46]. It may also happen that the *p*-values reported in a publication deviate from the *p*-value calculated from the data. However, it is curious that the incidence of these errors increases with journal rank, and the errors universally lower the *p*-value, rather than increase it, as one would expect if these errors were due to chance alone [42]. In the arms race between authors desperate to get ahead of the competition and journals trying to detect questionable research practices, the low-hanging fruit seem to be collected by the high-ranking journals: the rate of duplicated images is lower in these journals than in other journals [47]. This constitutes the currently only, to my knowledge, example in the literature in which higher-ranking journals appear to be better at catching errors than lower-ranking journals, as the lone exception to the rest of the literature.

These few examples stand in for a growing body of evidence in which high-ranking journals seem to often struggle to reach even average reliability [8,48]. In fact, some of the most convincing studies point towards an inverse relation between journal rank and reliability. A straightforward ad hoc hypothesis explaining this observation is that the emphasis of editors on novelty increases with journal rank, but editorial focus on scientific rigor and reliability does not. Given that novel and surprising results ought to be met with increased scrutiny, the data seem to suggest that this increase in editorial and statistical scrutiny does not take place. Taken together, the available evidence therefore not only invalidates the current use of IF specifically and of our subjective journal rank more generally but also demonstrates how counterproductive their deployment in evaluations proves to be by rewarding unreliable science.

This body of evidence points in the same direction as complementary research showing that selecting researchers based on their productivity also leads to decreased reliability [49,50]: selecting scientists on number of publications and journal rank will, over time, tend to decrease scientific reliability. In both cases, scientists are hired and promoted who publish less reliable work than their peers and who then go on and teach their students how to become successful scientists—by publishing a lot and in prestigious journals. This research is agnostic to the intention or motives of the individuals. Training, strategies, and competence all vary among the population of early career researchers from which institutions hire faculty. Using the common selection criteria ensures a bias towards unreliability, irrespective of its ultimate underlying source or reason, and institutions employ them at their own risk. Therefore, inasmuch as the number and venue of scholarly publications are used as measures for scientific “excellence,” the currently available data support recent parallel conclusions that this “excellence” is not excellent [51]. As it stands, “used in its current unqualified form it is a pernicious and dangerous rhetoric that undermines the very foundations of good research and scholarship” [51].

## Potential solutions

Although a case can be made for rewarding novel, risky discoveries over incremental, reliable advances in general, striking a balance between novelty and reliability is not trivial. Consistently rewarding novelty at the cost of reliability may prove counterproductive in the long term. Permanent positions for publicly funded researchers ought to be reserved for scientists who have earned the privilege to be entrusted with public funds by producing reliable science that is also novel. From these arguments and the available data, a number of potential evidence-based solutions can follow. Inasmuch as there is a common desire to signal novelty to a wider audience than the directly relevant scholarly peers, all these solutions would need to include a separation of such a novelty signal from a reliability signal if the overarching goal is to maintain—or even potentially increase—the reliability of science.

### Eliminate journal rank in evaluations

One of the standard solutions read and heard most often is to eliminate journal rank in any evaluation setting, as proposed by, e.g., signatories of the Declaration of Research Assessment (DORA) [52]. However, although activism in this direction targets the means by which unreliability is rewarded, the behavior of authors and editors underlying the reduced reliability in high-profile journals is unlikely to change until the abandonment of journal rank is near universal. This task can take decades, given that over 7 million full-time equivalent researchers would need to be convinced [53]. Moreover, with the high correlation between community assessment of journal quality and IF [32–36], it is safe to assume that scholarly communities will treat publications in certain venues differently, if only informally, even after such a universal ban on journal rank in evaluations were ever achieved. Such a solution, although likely to be effective in principle, is equally likely to take decades to achieve a noticeable effect.

### How much time do we have?

In the light of a prominent political persuasion currently in government in the United States recommending “when you hear a scientist talk about ‘peer-review’, you should reach for your Browning” [54] (an adapted quote from a 1933 play in the honor of Adolf Hitler, no less) and asking “do we really need government-funded research at all?” [55], it would appear self-defeating to not address systemic factors inflating scientific unreliability as soon as possible. With parties of similar persuasion already in power in Brazil, Hungary, Poland, Argentina, Austria, and Italy—or dominating political discourse in countries such as Sweden or Germany, and ever only an election cycle away from government in many other countries—it would seem wise to not provide arguments for the destruction of publicly funded science. Although it may be impossible for scholars alone to stop an antiscience ideology, there is little reason to help and support the enemies of science along their way. One may even question what good well-intended attempts to quantify the current reliability of science (e.g., replication projects) could do, when all the available evidence points towards unreliability being favored over reliability for decades already. With the evidence revealing a downward trajectory, isn’t it our duty as citizens to try to reverse the trend no matter how bad it has already gotten, and to do so quickly, to prevent further erosion?

### Eliminate journals

A faster solution would be to eliminate the source of evaluation by journal rank—journals. The vast majority of our over 30,000 peer-reviewed journals are currently funded via subscriptions. With an average duration of a subscription contract ranging from one to three years, the defunding and, consequently, elimination of journals could start as soon as next year and may be nearing completion as soon as three years from now.

There is no dearth of modern solutions that will improve quality control, curation, filtering, and discovery once scholarly publishing is aligned with a modern workflow without journals and divorced from novelty assessments. As such solutions have been proposed before and largely converge [56–58], I will not elaborate on them in detail. Recently in the companion article, Stern & O’Shea [70] suggested another, very attractive solution for such an infrastructure with sparser, more effective peer-review. In brief, a combination of existing versioning and badging technologies serves to mark the state of the publication at hand, e.g., working paper, peer-reviewed, data and statistics tested, replicated, in clinical trials, etc. With a modern infrastructure that curates, archives, and makes accessible all scholarly works, not just text-based narratives, we are free to reward scholars for other contributions. This opportunity alone will reduce the pressure to outperform the competition via text-based narratives, but it will also allow us to directly reward, e.g., the teaching of reproducible science and reproducing important findings.

Why would libraries start to cancel their subscriptions? For one, they are already canceling “Big Deal” subscriptions for various reasons of their own [59]. Moreover, their scholars may ask them for more modern infrastructure, because they need it for their work. However, scholars may also ask for modern infrastructures from their institutions because of prerequisites for research grants. Funding agencies may require institutions to implement available modern digital infrastructure before research grants can be awarded. Such criteria already exist in most funding agencies and would only need to be more specific and more strictly enforced, e.g., by a certification process. In addition to such basic infrastructure requirements, many funders also already support such a transition either by implementing their own publishing platforms [60] and/or by mandating author behavior, e.g., PlanS [61]. A certification to ensure institutions have implemented the infrastructure necessary for their grant recipients to be able to comply with such funder mandates thus seems like a small step from current practice. Moreover, many institutions already have implemented the first, initial components for such an infrastructure, e.g., databases and various types of “green,” subject, or institutional repositories on which they could build. Such a plan for requiring a modern infrastructure has been called “Plan I” (for infrastructure).

There are three main reasons for institutions to use subscription funds to pay for the required infrastructure and its certification. First, subscriptions are, by now, all but obsolete: most institutions retain archival rights to once subscribed content via various means. In addition, more and more technologies (e.g., Unpaywall, R4R, Kopernio) provide fully legal access to nominally paywalled new articles (and for the shrinking rest during the short transition, there still is Sci-Hub). Second, the new infrastructure performs all the functions of journals, only more effectively and with better functionality. Third, because modern article publishing is at least one order of magnitude cheaper than subscription-based publishing [62–66], the institutions stand to save significant amounts of money.

Therefore, subscription funds are more than sufficient to not only keep scholarly publishing going uninterrupted but also to implement the infrastructure required by funders. Ideally, the infrastructure would be decentralized, federated, and implemented under the governance of the scientific community [67]. Besides publishing our texts, such a solution would not only solve current problems establishing findable, accessible, interoperable, and reusable (FAIR) [68] infrastructures for research data as well as scientific source code and/or software and save taxpayers billions every year, it would also help separate novelty from reliability signals.

Because such an infrastructure would make data and code automatically (i.e., without extra work for scholars) accessible with the article, reliability tests can be performed more easily and more quickly. With novelty assessments separated from reliability assessments, tests for reliability can be applied differentially, according to need, rather than across the board as it is deployed now. Current practice treats peer-review like an unlimited resource. Inasmuch as peer-review can be effective at all, it would be wise to rather administer it sparsely, where it is most effective and most necessary (see also Stern & O’Shea [70]). There are many ways in which such sparse, selective allocation can be realized [56–58], and an infrastructure under the governance of the scholarly community would allow us to find out which is the best one of them.

### Eliminate hypercompetition

As some of the main drivers behind unreliability in science are thought to be socioeconomic, an alternative solution would be to eliminate the hypercompetition and the resulting stratified environment affecting most scholars today. However, it appears that this option both is more difficult to achieve than any of the solutions suggested above and lies partially outside the jurisdiction of scholars themselves. If it is impossible to eliminate this competition, the least we can do is strive to mitigate its negative consequences.

## “Too long; didn’t read”

There is a growing body of evidence against our subjective notion of more prestigious journals publishing “better” science. In fact, the most prestigious journals may be publishing the least reliable science. Therefore, it may not be pure coincidence that, in the fields in which the hierarchy of journals is playing an outsize role in rewarding scholars, the replication of scientific findings, or the lack thereof, is receiving more and more attention. Abandoning the expensive anachronism of journals may not only allow us to regain control over the important scholarly communications infrastructure and refocus it towards reliability, but it will also free sufficient funds to implement current technologies that will service our research data and scientific code and/or software such that, e.g., biological databases would never face money-related closures again. Funders may play an important role in the transition from the legacy to the modern system in that they could require the institutions of grant applicants to join the modern system before any applications are reviewed (i.e., a “Plan I”, for infrastructure).

## Abbreviations

CRISPR: clusters of regularly interspaced short palindromic repeats
DORA: Declaration of Research Assessment
FAIR: Findable, accessible, interoperable and re-usable
IF: impact factor
MMR: mumps, measles, rubella
